# Altered Striatocerebellar Metabolism and Systemic Inflammation in Parkinson's Disease

**DOI:** 10.1155/2016/1810289

**Published:** 2016-09-01

**Authors:** Chiun-Chieh Yu, Meng-Hsiang Chen, Cheng-Hsien Lu, Yung-Cheng Huang, Hsiu-Ling Chen, Nai-Wen Tsai, Hung-Chen Wang, I-Hsiao Yang, Shau-Hsuan Li, Wei-Che Lin

**Affiliations:** ^1^Department of Diagnostic Radiology, Kaohsiung Chang Gung Memorial Hospital and Chang Gung University College of Medicine, Kaohsiung 83305, Taiwan; ^2^Department of Neurology, Kaohsiung Chang Gung Memorial Hospital and Chang Gung University College of Medicine, Kaohsiung 83305, Taiwan; ^3^Department of Nuclear Medicine, Kaohsiung Chang Gung Memorial Hospital and Chang Gung University College of Medicine, Kaohsiung 83305, Taiwan; ^4^Department of Neurosurgery, Kaohsiung Chang Gung Memorial Hospital and Chang Gung University College of Medicine, Kaohsiung 83305, Taiwan; ^5^Department of Internal Medicine, Kaohsiung Chang Gung Memorial Hospital and Chang Gung University College of Medicine, Kaohsiung 83305, Taiwan

## Abstract

Parkinson's disease (PD) is the most second common neurodegenerative movement disorder. Neuroinflammation due to systemic inflammation and elevated oxidative stress is considered a major factor promoting the pathogenesis of PD, but the relationship of structural brain imaging parameters to clinical inflammatory markers has not been well studied. Our aim was to evaluate the association of magnetic resonance spectroscopy (MRS) measures with inflammatory markers. Blood samples were collected from 33 patients with newly diagnosed PD and 30 healthy volunteers. MRS data including levels of N-acetylaspartate (NAA), creatine (Cre), and choline (Cho) were measured in the bilateral basal ganglia and cerebellum. Inflammatory markers included plasma nuclear DNA, plasma mitochondrial DNA, and apoptotic leukocyte levels. The Cho/Cre ratio in the dominant basal ganglion, the dominant basal ganglia to cerebellum ratios of two MRS parameters NAA/Cre and Cho/Cre, and levels of nuclear DNA, mitochondrial DNA, and apoptotic leukocytes were significantly different between PD patients and normal healthy volunteers. Significant positive correlations were noted between MRS measures and inflammatory marker levels. In conclusion, patients with PD seem to have abnormal levels of inflammatory markers in the peripheral circulation and deficits in MRS measures in the dominant basal ganglion and cerebellum.

## 1. Introduction

Parkinson's disease (PD) is the second most common neurodegenerative disease after Alzheimer's disease and has been characterized as a progressive neurological disorder without any available cure or preventative treatment [1, 2]. The etiology of PD remains uncertain. Genetic and exogenous factors (such as aging, environmental factors, and oxidative stress) and their interaction play key roles in PD pathogenesis [1, 3]. Neuroinflammation has been considered to be closely related to the progression of PD [4]. Elevated oxidative stress and mitochondrial involvement may trigger neuroinflammation [5, 6], thereby activating microglia and astrocytes and facilitating subsequent infiltration of the blood brain barrier (BBB) by peripheral immune cells [7, 8]. Imbalance in cellular homeostasis leads to different forms of programmed neuron cell death including apoptosis and necrosis [9–11]. Moreover, previous studies show leukocyte apoptosis is significantly higher in PD patients and associated with central dopamine neuron loss [12]. Demonstration of an association between peripheral and central inflammation might help clarify the pathogenesis of PD.

Proton (^1^H) magnetic resonance spectroscopy (MRS) can be used to noninvasively monitor changes in brain metabolite levels in PD patients, and methods of detecting levels of specific hydrogen-containing compounds* in vivo* can be used to measure neuron cell integrity and brain energy metabolism in PD patients [13]. Metabolites measured by ^1^H-MRS include N-acetylaspartate (NAA; a marker of neuronal integrity), choline-containing compounds (Cho; a marker of membrane turnover and glial proliferation), and creatine with phosphocreatine (Cre; a marker of tissue energetic metabolism, usually considered an internal control). MRS is widely used to monitor brain areas including the basal ganglia, substantia nigra, temporoparietal cortex, prefrontal cortex, posterior cingulated cortex, pontine basis, and occipital lobe [13–15] and as a diagnostic tool for differentiating PD from other neurodegenerative diseases [16]. Recently, it has been demonstrated that PD's effect on the striatopallidal circuit and cerebellothalamocortical circuit is responsible for many clinical features [17, 18]. As PD progresses, there is a gradual increase in the severity of motor dysfunction (including resting tremor, akinesia, bradykinesia, and rigidity indicating impaired coordination of the motor cortex and cerebellum) [19]. The metabolic change in these two circuits in PD patients is not well evaluated by MRS.

PD is diagnosed anatomopathologically and a definite diagnosis can only be reached postmortem. This study hypothesized that MRS findings reflect neuroinflammatory changes in PD and are associated with peripheral inflammation. For this reason, we statistically evaluated the correlation of brain metabolite levels (measured by MRS) with selective peripheral inflammatory biomarker levels and disease severity. The results not only link peripheral inflammation with neuroinflammation in PD patients but also suggest that inflammation assessed by both imaging and molecular biological methods could be used to monitor disease progression.

## 2. Materials and Methods

### 2.1. Participants

The study protocol conformed to the ethical guidelines of the 1975 Declaration of Helsinki as reflected in à priori approval by the Chang Gung Memorial Hospital Human Research Committee. Thirty-three patients (22 males and 11 females, mean age 59.8 ± 9.24 years) with idiopathic PD diagnosed according to United Kingdom Brain Bank criteria [20] by an experienced neurology specialist and without histories of other neurologic or psychiatric illnesses and psychotropic medications were prospectively enrolled at the Neurology Department of Chang Gung Memorial Hospital and included. In each case, the time of diagnosis and the duration of the disease were recorded. Disease onset was defined as the time of first recalled motor symptoms, such as tremor, bradykinesia, and rigidity in the pretreatment phase of the disease. Patients with systemic diseases including cancer and end-stage kidney disease under hemodialysis were excluded.

The studies were performed at least 12 h after the last dose of dopaminergic medication (off state). Disease severity and functional status were evaluated using the Mini-Mental State Examination (MMSE) [21], Unified Parkinson's Disease Rating Scale (UPDRS), modified Hoehn and Yahr staging scale (HY-stage), and Schwab and England Activities of Daily Living Scale (SE-ADL). The Mini-Mental State Examination (MMSE) is used to assess general cognitive function. The UPDRS score is used to follow the longitudinal course of PD [22] and is evaluated by interview and clinical observation. The modified HY-staging scale is used to globally assess the severity of Parkinson's disease based on clinical findings and functional disability [23] and to describe the progression of Parkinson's disease from HY-stage 1 through HY-stage 5. The SE-ADL is used to assess functional abilities (relative independence), with 100% indicating complete independence and 0% indicating total dependence.

For comparison, 30 sex- and age-matched healthy subjects (17 males and 13 females; mean age 57.0 ± 9.58 years) without a medical history of neurologic disease or psychiatric illness, alcohol or substance abuse, or head injury and with similar levels of education were recruited from the hospital as the control group. The hospital's institutional review committee on human research approved the study protocol, and all of the participants or their guardians provided written informed consent.

### 2.2. MR Spectroscopy Acquisition

**Figure 1:**
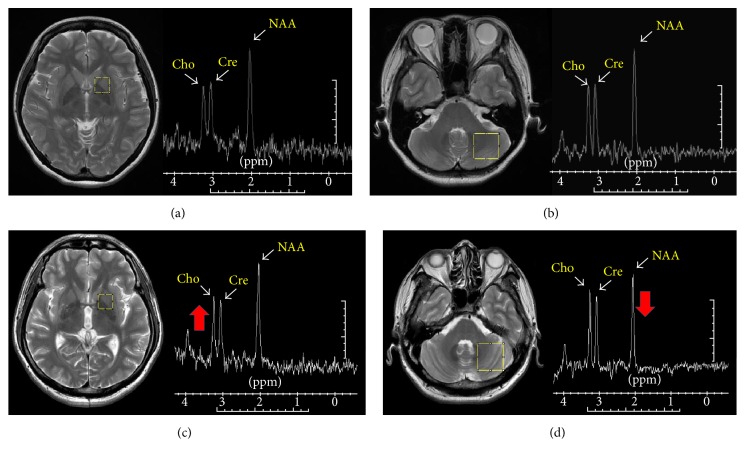
Location of the regions of interest and corresponding representative spectra. Region of interest in (a) basal ganglia, (b) cerebellum of a healthy volunteer and (c) basal ganglia, and (d) cerebellum of a PD patient. Major peaks in representative spectrum indicate choline (Cho), creatine (Cre), and N-acetylaspartate (NAA). The metabolite Cho peak increase in basal ganglia of PD (c) and NAA peak decrease in cerebellum of PD patient (d) (indicated by thick arrow).

The MRS protocol was performed on a GE Signa 3T whole-body MRI scanner (General Electric Healthcare, Milwaukee, WI, USA) with an eight-channel head coil. Axial FSE T2-weighted images (repetition time, TR: 4200 ms, echo time, TE: 102 ms, and 2.5 mm slice thickness) and T1-weighted structural images of the whole head using the 3D-FSPGR sequence (repetition time [TR] = 9.492 ms, echo time [TE] = 3.888 ms, flip angle 20°, field of view [FOV] = 24 × 24 cm, matrix size = 512 × 512, 110 continuous slices with the slice thickness of 1.3 mm, and in-plane spatial resolution of 0.47 × 0.47 mm) were acquired to optimize the localization. Data acquisition was performed using point resolved spectroscopy (PRESS) sequences with a repetition time (TR)/echo time (TE) of 1500/144.0 ms and 128 acquisitions. Suppression of the water signal was adjusted before data collection using a frequency selective double inversion recovery sequence. Field homogeneity was shimmed to give a full width at half maximum (FWHM) less than 10 Hz before data acquisition, and the percentage of water suppression higher than 95% was used to minimize the influence of signal-noise-ratio. Data were collected from a 1.5 × 1.5 × 2.0 cm^3^ voxel of interest (VOI) located within the basal ganglia and 2.4 × 2.4 × 2.4 cm^3^ VOI located within the cerebellum (Figure 1). Because PD is regarded as an asymmetric movement disorder, we defined the dominant side as the side with the highest UPDRS score and most affected by clinical symptoms and the nondominant side as the contralateral side. All spectra were first visually assessed in a blinded fashion by an experienced investigator. Spectra were evaluated for general quality, which was dependent on noise level, baseline, water suppression, and ability to identify 3 major metabolic peaks. Spectra were postprocessed by means of manufacturer-provided software (SAGE; General Electric Medical Systems, Milwaukee, WI). Curve fitting and line width normalization were performed, and 3 major peaks corresponding to NAA (2.0 ppm), Cre (3.0 ppm), and Cho (3.2 ppm) were identified. The total time needed to examine the MRI and ^1^H-MRS scans was less than 1 h.

### 2.3. Basal Ganglia-Cerebellum Ratio of MRS Parameters

The ratios of NAA and Cho relative to Cre were used to minimize the influence of structural tissue atrophy within the VOI on the target image [24]. The ratios of NAA relative to Cho and (Cho + Cre) were also used for comparison in a previous study [25]. Furthermore, taking into account the fact that the brain coordinates communication, the striatum-cerebellum ratio was calculated by dividing NAA/Cre (for the dominant and or nondominant basal ganglia) by the NAA/Cre (for the cerebellum). Moreover the striatum-cerebellum ratios for Cho/Cre, NAA/Cho, and NAA/(Cho + Cre) in these two regions were also calculated.

### 2.4. Laboratory Measurement of Inflammatory Markers in the Peripheral Circulation

#### 2.4.1. Blood Sampling

Oxidative stress was evaluated in all subjects in terms of percentage of apoptotic peripheral leukocytes and plasma levels of nuclear and mitochondrial DNA. Blood was drawn by venipuncture from the forearm on the same day as the MRI study and neuropsychological testing.

#### 2.4.2. Assessment of Leukocyte Apoptosis

Procedural details were as described previously [12]. Whole blood (100 *μ*L) was stained with 10 *μ*L of CD45-phycoerythrin- (PE-) Cy5 antibody (clone J33) for 15 min at room temperature and protected from light. The CD45-PE-Cy5 antibody reacts with the CD45 family of transmembrane glycoproteins, expressed on the surface of all human leukocytes, and is a pan-leukocyte marker. Cells were fixed with 5.5% formaldehyde, washed, exposed to a permeability-inducing agent (Beckman Coulter Inc., Fullerton, CA), exposed to a hypotonic solution to lyse the remaining erythrocytes, and incubated with APO 2.7-PE (clone 2.7A6A3; Immunotech, Marseille, France) to tag the 38-kDa mitochondrial membrane protein (7A6 antigen), which is detectable on nonpermeabilized cells in the late apoptotic stage [26]. Mouse immunoglobulin G-PE was used as a control for nonspecific staining. The leukocytes were then analyzed by flow cytometry.

Immediately after staining, flow cytometry was performed on an Epics XL flow cytometer (Beckman Coulter, Fullerton, CA) and the data analyzed using EXPO32 ADC software. Five thousand CD45-PE-Cy5+ cells per sample were acquired in a combined forward-and-side scatter and deep-red FL4 fluorescence (CD45-PE-Cy5) leukocyte gate. Leukocyte subtypes were identified according to their CD45 expression intensity. The results were expressed as the percentage of specific fluorescence-positive cells. Apoptotic cells were defined by APO 2.7 positivity. A database coordinator was responsible for monitoring all data collection and entry. All data were checked for any inconsistencies. Intra-assay variability based on repeated measurements of the same blood sample was low.

#### 2.4.3. Determination of Plasma Nuclear DNA and Mitochondrial DNA Levels

In every patient, 3 mL of peripheral venous blood was collected into ethylenediaminetetraacetic acid-containing tubes. Procedural details were as described previously [27, 28]. To ensure cell-free specimen collection, the blood samples were initially centrifuged for 10 minutes at 3000 ×g and the plasma was transferred to 1.5 mL clear polypropylene tubes (with care not to disturb the buffy coat layer) and centrifuged for extra 10 minutes at 16,000 ×g. The upper portion of the plasma was removed with a Pasteur pipette (approximately 200 *μ*L), placed into another set of clear tubes, and frozen at −20°C prior to extraction. The DNA was extracted from plasma samples using a QIAamp Blood Kit (Qiagen, Hilden, Germany) according to the manufacturer's blood and body fluid protocol. Per column, 200 *μ*L of plasma sample was used for DNA extraction, and the exact amount used was documented to enable calculation of the target DNA concentration.

The plasma nuclear DNA was measured by a real-time quantitative polymerase chain reaction (RT-PCR) assay (Roche LightCycler, Roche, Grenzach-Wyhlen, Germany) for the *β-globin. β-globin* is a kind of housekeeping gene detected in plasma free circulating DNA associated with disease and as a tool to analyze fetal DNA in mother plasma and serum [27]. Each run (including a standard curve, “positive” genomic DNA control, and “negative” [deionized water] control) was repeated. The PCR system for the *β-globin* gene (which is present in all nucleated cells of the body as well as plasma nuclear DNA) consisted of the amplification primers *β*-globin-354F (5′-GTGCACCTGACTCCTGAGGAGA-3′) and *β*-globin-455R (5′-CCTTGATACCAACCTGCCCAG-3′). Expression of *β-globin* gene in plasma nuclear DNA was measured by continuous monitoring of SYBR green fluorescent dye incorporation into the double-stranded DNA product of PCR.

*ND2* gene is core subunit of the mitochondrial membrane respiratory chain NADH dehydrogenase (complex I) and used as a marker for mitochondrial DNA. The PCR system for the* ND2* gene was the specific primer pair for ND2 (forward 5′-CACAGAAGCTGCCATCAAGTA-3′, reverse 5′-CCGGAGAGTATATTGTTGAAGAG-3′). Expression of* ND2* gene in free circulating mitochondria DNA was measured using the above quantitative RT-PCR system [28]. The DNA standard curve was generated using human genomic DNA (Roche). The previously reported imprecision of this system (i.e., coefficient of variation of the threshold cycle) was 1.1%. Quantitative results are expressed as ng/mL.

### 2.5. Statistical Analysis

The demographic data, including age and sex, were compared among the study groups by the 2-sample Student* t*-test or the Mann-Whitney test, where appropriate, and are reported as mean ± the standard deviation (SD) or median (interquartile range). The significance of differences in inflammatory markers, disease severity, and MRS variables was determined by analysis of covariance (ANCOVA) with the participant's age and sex as covariates. The associations between inflammatory markers and MRS variables were tested by partial correlation analysis after adjustments for age and sex. The threshold for all statistical significance was set at *P* < 0.05. SPSS software (SPSS V.12, Chicago, IL, USA) was used to perform all statistical analyses.

## 3. Results

### 3.1. Baseline Characteristics of the Study Patients

While both the patient and control groups (Table 1) had similar age and sex distribution, the control group had significantly higher Mini-Mental State Examination (MMSE) scores. Clinical disease severity on the Unified Parkinson's Disease Rating Scale (UPDRS-I, UPDRS-II, and UPDRS-III), modified HY-stage scale, and SE-ADL scale could only be evaluated in the patient group.

### 3.2. MR Spectroscopy for Brain Metabolite Measurement

#### 3.2.1. MRS Findings in the Dominant Basal Ganglia

**Figure 2:**
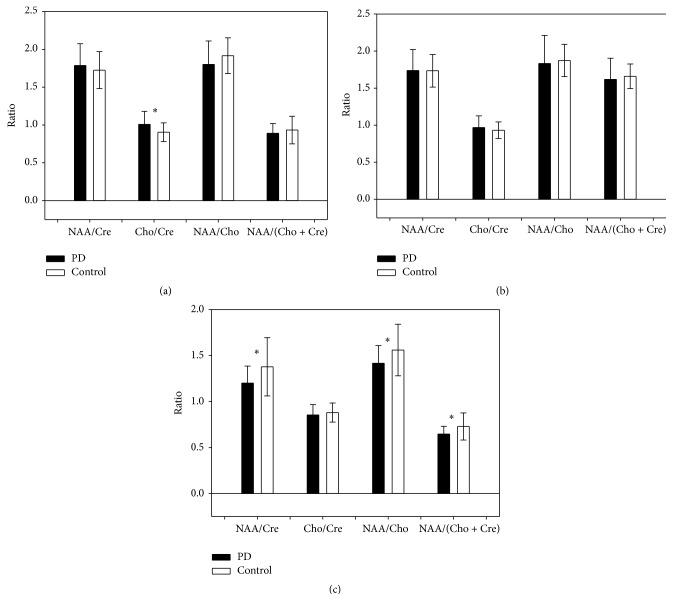
Comparison of brain metabolite ratios in the (a) dominant basal ganglia, (b) nondominant basal ganglia, and (c) cerebellum in patients with Parkinson's disease and control subjects. PD: Parkinson's disease. ^*∗*^ *P* < 0.05, PD patients versus controls. Error bars reflect standard deviation (SD).

^1^H-MRS showed a significant between-group difference in Cho/Cre (i.e., 12% higher in the dominant basal ganglia of the PD group than in the basal ganglia of the control group [1.07 ± 0.17 versus 0.9 ± 0.12; *P* = 0.006; Figure 2(a)]) but not in NAA/Cre (1.79 ± 0.29 versus 1.73 ± 0.24), NAA/Cho (1.80 ± 0.31 versus 1.92 ± 0.24), or NAA/(Cho + Cre) (0.89 ± 0.13 versus 0.93 ± 0.18).

#### 3.2.2. MRS Findings in the Nondominant Basal Ganglia

There was no significant difference in NAA/Cre, Cho/Cre, NAA/Cho, or NAA/(Cho + Cre) (Figure 2(b)) between the nondominant basal ganglia of the patient group and basal ganglia of the control group (i.e., 1.74 ± 0.28 versus 1.73 ± 0.22; 0.97 ± 0.16 versus 0.93 ± 0.11; 1.83 ± 0.38 versus 1.87 ± 0.22; 1.62 ± 0.28 versus 1.66 ± 0.17, resp.).

#### 3.2.3. MRS Findings in the Cerebellum

The cerebellar NAA/Cre, NAA/Cho, and NAA/(Cho + Cre) ratios were significantly decreased in the patient group (1.20 ± 0.18 versus 1.38 ± 0.32 [*P* = 0.018], 1.42 ± 0.19 versus 1.56 ± 0.28 [*P* = 0.023], and 0.65 ± 0.08 versus 0.73 ± 0.15 [*P* = 0.015], resp.; Figure 2(c)) but the cerebellar Cho/Cre ratio was not affected (0.85 ± 0.11 versus 0.88 ± 0.10).

#### 3.2.4. The Basal Ganglia to Cerebellum Ratio of MRS Parameters

**Figure 3:**
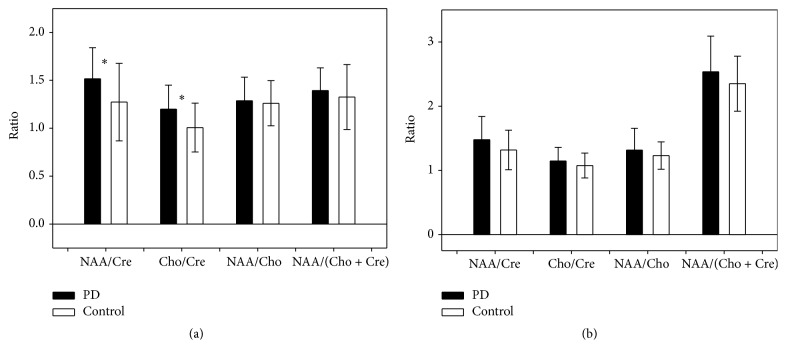
Comparison of the (a) dominant basal ganglion-cerebellum and (b) nondominant basal ganglion-cerebellum ratios for the MRS parameters (NAA/Cre, Cho/Cre, NAA/Cho, and NAA/[Cho + Cre]) in patients with Parkinson's disease and control subjects. PD: Parkinson's disease. ^*∗*^ *P* < 0.05, PD patients versus controls. Error bars reflect standard deviation (SD).

Figures 3(a) and 3(b) show that the dominant basal ganglia to cerebellum ratios of two MRS parameters NAA/Cre and Cho/Cre were significantly different between the two groups (1.52 ± 0.32 versus 1.27 ± 0.40 [*P* = 0.019] and 1.20 ± 0.25 versus 1.01 ± 0.25 [*P* = 0.004]).

### 3.3. Percent of Apoptotic Leukocytes, Plasma Nuclear DNA Level, and Mitochondrial DNA Level

**Figure 4:**
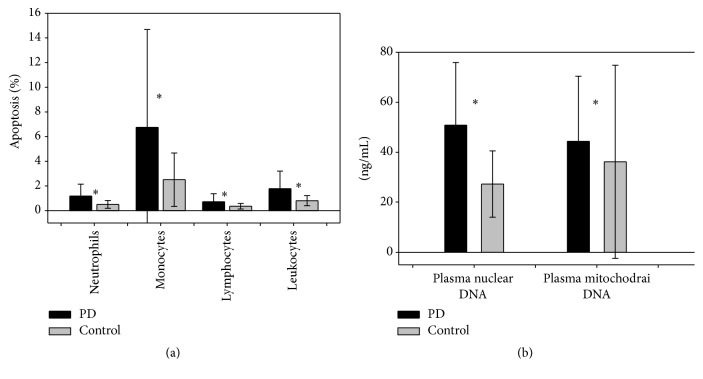
Oxidative biomarkers in patients with Parkinson's disease and control subjects. (a) Apoptotic leukocytes and apoptotic leukocyte subpopulations (percentage) and (b) levels of plasma nuclear DNA and mitochondrial DNA. PD: Parkinson's disease. ^*∗*^ *P* < 0.05, PD patients versus controls. Error bars reflect standard deviation (SD).

Analysis of the inflammatory marker data, presented as median value (interquartile range), showed that PD patients had significantly higher percentage of leukocyte apoptosis (1.26 [0.79, 2.09] versus 0.77 [0.46, 0.95]), neutrophil apoptosis (0.81 [0.56, 1.47] versus 0.48 [0.26, 0.60]), monocyte apoptosis (3.85 [2.28, 7.62] versus 1.79 [1.27, 3.35]), lymphocyte apoptosis (0.55 [0.35, 0.76] versus 0.32 [0.21, 0.50]) (Table 1) (Figure 4(a)), and plasma nuclear DNA and mitochondrial DNA levels (55.70 [27.20, 72.50] versus 25.45 [20.35, 33.68]) and (36.00 [28.40, 43.00] versus 23.80 [6.50, 39.70]) (Table 1) (Figure 4(b)).

### 3.4. Correlations of Blood Oxidative Stress Markers and Brain Metabolites on MRS with Disease Severity

The basal ganglion-cerebellum ratio of the MRS parameter Cho/Cre was correlated with plasma nuclear DNA level and percentage of apoptotic leukocytes (total), neutrophils, and lymphocytes (*r*,  *P* = 0.384, 0.01; 0.271, 0.045; 0.376, 0.005; 0.422, 0.001, resp.; Tables 2 and 3), and the Cho/Cre ratio in the dominant basal ganglia was correlated with increased percentage of apoptotic total leukocytes, neutrophils, and lymphocytes (*r*,  *P* = 0.277, 0.041; 0.313, 0.02; 0.366, 0.006, resp.; Table 2). Moreover, the basal ganglion-cerebellum ratio of NAA/Cre also correlated with the percentage of apoptotic neutrophils (*r*,  *P* = 0.299, 0.027; Table 2). However, disease severity was not correlated with plasma nuclear DNA level, apoptotic leukocyte percentage, or MRS parameters.

## 4. Discussion

In this study, NAA/Cre was significantly decreased, while Cho/Cre was significantly increased in the dominant basal ganglion and the cerebellum (compared with healthy basal ganglia and cerebellum), suggesting the occurrence of neuron death and glial cell proliferation as sequelae of neuroinflammation. Furthermore, changes in the level of circulating plasma DNA and apoptotic leukocyte percentage and leukocyte subpopulation percentages reinforce the notion of peripheral inflammation. The correlations of MRS parameters in the dominant basal ganglion and cerebellum and the basal ganglion-cerebellum ratio with circulating plasma nuclear DNA level and apoptotic leukocyte percentage may mirror the relationship between neuroinflammation and peripheral inflammation during the neurodegenerative process in PD.

### 4.1. The Change in MRS Parameters Reflects the Change in Brain Metabolites

Neuroinflammation plays a key role in the pathogenesis of Parkinson disease, and inflammatory markers like C-reactive protein (CRP) and monocyte chemotactic protein-1 (MCP-1) in cerebrospinal fluid (CSF) were demonstrated to be associated with nonmotor features of PD [29]. Unlike CSF sampling, MRS is noninvasive and provides a different method of measuring brain metabolite change and of demonstrating another pattern of neuroinflammation [30].

In our MRS study, we targeted the basal ganglia and cerebellum. The levels of brain metabolites including NAA, Cho, and Cre were measured and the MRS parameter Cho/Cre was significantly elevated in the dominant basal ganglion. The similarity in the basal ganglion NAA/Cre ratio between PD patients and controls is consistent with previous findings [14, 15]. A previous small cohort study revealed no difference in the Cho/Cre ratio of the lentiform nucleus and caudate between PD and normal subjects [31]. But another study showed an elevated choline level in the caudate nucleus [32]. The elevation in basal ganglion Cho/Cre indicates not only glial cell proliferation but also the possibility of soluble Cho release from degenerating cholinergic neurons [14]. In a previous pilot study, the NAA/Cho ratio in the striatum region was similar between patient and control groups of all ages (27–83 years) but significantly decreased in an elderly subset of patients (51–70 years). The study also reported that NAA/Cho ratios in the striatum region were significantly below normal in drug-naïve PD patients but normal in levodopa-treated patients and controls [33]. These data suggest that dopaminergic treatment may affect NAA levels in the striatum of PD patients and that the similarity in NAA/Cho ratio of the basal ganglia between the PD patients and control group in our study may be due to influence of dopaminergic treatment.

In the cerebellum, NAA/Cre, NAA/Cho, and NAA/(Cho + Cre) were decreased and suggest MRS is a potential tool for diagnosis of PD. The advantage of using the cerebellum to obtain an accurate metabolic profile is that (because of its relatively large size) the partial volume effect of adjacent structures can be avoided [19]. The results of the present study demonstrate that PD affects the parts of the basal ganglia and cerebellum involved in PD-related motor symptoms.

To further identify the coordination between basal ganglia and cerebellum using MRS, the basal ganglia-cerebellum ratio was calculated and the significance of this ratio for the MRS parameters NAA/Cre and Cho/Cre was noted. Actually, basal ganglia and cerebellum have distinct anatomically demonstrable circuits that connect with largely overlapping cortical areas [18] and integrate both the motor and nonmotor domains of basal ganglia and cerebellar function [19]. As the disease progresses, brain areas including the striatopallidal and cerebellothalamocortical networks gradually become dysfunctional [17, 18]. Voxel-Based Morphometry (VBM) indicated significant cerebellar GM atrophy and loss of functional connectivity within cerebellar-cortical networks. The influence of the subthalamic nucleus on intracerebellar connectivity is also lost [34]. In this study, the calculated basal ganglia-cerebellum ratios of MRS parameters (NAA/Cre and Cho/Cre) were significantly larger in the PD group than in controls, indicating asymmetric degeneration of these two brain regions and the possibility that the basal ganglia-cerebellum ratio of NAA/Cre or Cho/Cre could be another noninvasive biomarker of PD progression.

### 4.2. Leukocyte Apoptosis Percentage as a Peripheral Inflammation Indicator in PD Patients

The significantly higher levels of circulating nuclear DNA, mitochondrial DNA, and apoptotic leukocytes in PD patients in the present study were important clues suggesting underlying peripheral inflammation contributing to neuroinflammation. In brain tissue, dysfunction of the BBB accompanied by peripheral immune cell infiltration/invasion leads to loss of dopaminergic neurons caused by programmed cell death [35, 36]. The cytokine tumor necrosis factor (TNF), a master regulator of the immune response, was demonstrated to play a major role in the propagation of inflammation through activation and recruitment of peripheral immune cells into the CNS [37].

In the CNS, oxidative damage is a critical factor accelerating the disease process, including mitochondrial DNA mutation and lysozyme dysfunction [38]. The accumulation of such mutations may lead to loss of mitochondrial DNA and thereby to a vicious cycle that generates more oxidative stress ending in programmed cell death (apoptosis) [39]. A recent review of the literature clarified the role of inflammatory and apoptotic cell death pathways in the brain and peripheral blood (monocyte apoptosis) of Parkinson's disease patients [40] and demonstrated that mitochondria copy number in peripheral blood was decreased and brain tissue (the substantia nigra pars compacta) was affected [41]. In the present study, levels of apoptotic leukocytes including neutrophils, lymphocytes, and monocytes were elevated in PD. In our previous study, the significant increase in leukocyte apoptosis in PD patients was associated with central dopamine neuron loss (as shown by ^99m^TcTRODAT-1 SPECT), suggesting that systemic inflammation correlates with neuroinflammation as this neurodegenerative disease progresses [12].

### 4.3. Elevated Plasma Nuclear DNA Level as a Clinical Manifestation of Neuroinflammation in PD Patients

The elevation of plasma nuclear DNA and mitochondrial DNA level in our PD patients suggested neuron loss is occurring due to a combination of neuron apoptosis and necrosis [42]. The influence of mitochondrial dysfunction may lead to an ionic imbalance, calcium overload, and ultimately ATP depletion. If ATP as an energy source dramatically decreases, necrotic cell death will ensue. In addition, excitotoxicity may account for neuron necrosis in acute brain injury as well as experimental models of stroke and Parkinson's disease [43]. Previous studies demonstrate that circulating plasma DNA is elevated in patients with inherited ataxia. This suggests that significantly higher concentration of plasma DNA may be due to neuronal and muscular degeneration in these patients [44] and possibly be used in the diagnosis, prognosis, and monitoring of status epilepticus [45] and aneurysmal subarachnoid hemorrhage [46].

Systemic diseases like cancer may increase levels of plasma DNA and decrease levels of mitochondrial DNA [47]. For this reason, patients with cancer were excluded to avoid contamination of the results. In this study, peripheral nuclear DNA elevation in the PD group supports the proposition that neuron death caused by neuroinflammation in the brains of PD patients can be detected by peripheral blood sampling. Other research has identified methylation patterns of circulating DNA [48] and circulating cell-free RNA [49] as indicators of tissue-specific cell death and as biomarkers for neurodegenerative disease.

### 4.4. Partial Correlation between MRS Parameters and Inflammatory Markers

The change in brain metabolites found in MRS studies may be a potential indicator of neuroinflammation [30] but more evidence is needed to prove it [50]. Previous studies have confirmed that peripheral inflammation increases the deleterious effect, contributes to further neurodegeneration [51], and drives disruption of brain networks [52]. For this reason, the combination of peripheral inflammatory markers with MRS findings can integrate more components of PD pathogenesis. After controlling for age and sex, the MRS parameter (Cho/Cre) in the dominant basal ganglion was weakly to moderately correlated [53] with the inflammatory marker (leukocyte apoptosis). Moreover, the basal ganglion-cerebellum ratio for the MRS parameter (Cho/Cre) was also weakly to moderately correlated with apoptotic leukocyte percentage and plasma nuclear DNA. The positive* weak to moderate* correlation may indicate an increasing trend toward a relationship between peripheral inflammatory markers and MRS findings [54]. In this study, leukocyte apoptosis elevation (peripheral inflammation) in PD patients was associated with the change in brain metabolite distribution due to neuroinflammation. The evidence for inflammatory and cell death pathways in the brain and peripheral blood in PD has been reviewed and reinforces the notion of neuroinflammation-peripheral inflammation interaction [40]. However, disease severity based on MRS findings and disease severity based on inflammatory markers were not correlated, consistent with previous MRS study in the basal ganglia [15]. The reason might have been the presence of statistical “ceiling” effects due to the selection of subjects [55, 56]. And small sample size with increased variability may influence the correlation between disease severity and magnetic resonance imaging finding [57]. Furthermore, disease severity scores present summation of motor and nonmotor deficits of PD. It is also well known that disease progression in PD reflected extensive brain damage and might not be simply related to specific brain region malfunction. Large sample size can provide more evidence and further investigate the relationship between clinical disease severity and biomarkers.

### 4.5. The Limitations of This Study

First, the effects of dopaminergic drugs on MRS are not well defined and a previous report showed NAA/Cho ratios in the putamen may be affected by L-dopa therapy [58]. Although our studies were performed at least 12 h after the last dose of dopaminergic medication (off state), this issue deserves further clarification. Second, brain circuit architecture reflects coordination between brain regions. The real brain network is very complicated but, in this study, we introduce the concept of brain region ratio (e.g., basal ganglion-cerebellum ratio) to reflect the asymmetry apparent on MRS. Indeed, the ratio may also suppress the true result. Moreover, one may ask the following question: is cerebellum a suitable target region for PD diagnosis? One previous study focused on MRS for differential diagnosis of PD syndromes with no significant difference in NAA/Cre ratio between the PD and healthy control group [57]. However, in our study, large VOI size and differences between MRI scanners (3T scanners) may influence the final results. Furthermore, involvement of the cerebellum in PD has been clarified using other brain magnetic resonance imaging techniques. Using diffusion tensor imaging (DTI) in the cerebellar hemispheres of PD revealed a fractional anisotropy (FA) decrease, reflecting white matter microstructure damage [59]. A larger sample size and further investigation of the relationship of particular brain networks and affected brain regions to MRS imaging findings are warranted. Third, although inflammatory and apoptotic cell death pathways in brain and peripheral blood (monocyte apoptosis) are known contributors to Parkinson's disease pathogenesis [40], monocyte apoptosis and proinflammatory activity also increase across CKD stages 1–4 [60]. Because renal function decreases with age, it is really difficult to select PD patients without any chronic kidney disease. In this study, we excluded PD patients with end-stage kidney disease under hemodialysis but still could not totally eliminate peripheral leukocyte apoptosis caused by impaired renal function. Forth, the limited strength of the correlation between oxidative stress markers and ratio of different brain metabolites in basal ganglia and cerebellum was observed. A holistic profile of brain metabolites of PD patients could certainly increase our understanding of neuroinflammation, neurotransmission, and brain metabolism [13, 61]. Further study can expand the profile of brain metabolites (such as alanine, aspartate, creatine, gamma-aminobutyric acid, glucose, glutamate, glutamine, glycerophosphorylcholine, phosphorylcholine, m-inositol, lactate, N-acetylaspartylglutamate, phosphocreatine, scyllo-inositol, and taurine) that can be measured by using MRS to make the diagnosis more robust. The concept “Metabolomics” provides a systematic approach to understand the pathology of PD by using magnetic resonance imaging to analyze brain metabolites [62].

## 5. Conclusions

MRS is a noninvasive method of detecting early PD via specific metabolite changes in the basal ganglia, cerebellum, and basal ganglion-cerebellum ratio. The MRS findings are correlated with elevated circulating plasma nuclear DNA and apoptotic leukocyte percentage and reflect the interaction of neuroinflammation with peripheral inflammation. The study demonstrates that inflammatory markers in peripheral blood combined with MRS findings in the brain can be used for early detection of PD. Further investigation may help uncover therapeutic targets for intervention.

## Figures and Tables

**Table 1:** Demographic data and inflammatory markers of PD patients and controls.

Parameters	PD patients (*n* = 33)	Controls (*n* = 30)	*P* value
Age (year) (mean ± SD)	59.80 ± 9.24	57.00 ± 9.58	0.239
Sex (M, F)	22, 11	17, 13	0.423
UPDRS I^#^	3.50 (2.00, 6.25)		
UPDRS II^#^	10.00 (6.00, 15.25)		
UPDRS III^#^	21.50 (17.00, 34.50)		
UPDRS total scores^#^	34.00 (25.75, 54.50)		
Modified HY-stage (maximum stage is 5)^#^	2.50 (1.00, 3.00)		
SE-ADL (minimum score 0 suggests presence of only vegetative function)^#^	80 (70, 90)		
MMSE^#^	24 (21, 28)	27 (26, 29)	0.021^**∗**^
*Inflammation parameters*			
Total apoptotic leukocytes (%)^#^	1.26 (0.79, 2.09)	0.77 (0.46, 0.95)	0.001^**∗**^
Apoptotic neutrophils (%)^#^	0.81 (0.56, 1.47)	0.48 (0.26, 0.60)	0.001^**∗**^
Apoptotic monocytes (%)^#^	3.85 (2.28, 7.62)	1.79 (1.27, 3.35)	0.016^**∗**^
Apoptotic lymphocytes (%)^#^	0.55 (0.35, 0.76)	0.32 (0.21, 0.50)	0.011^**∗**^
Nuclear DNA (ng/mL)^#^	55.70 (27.20, 72.50)	25.45 (20.35, 33.68)	0.002^**∗**^
Mitochondria DNA (ng/mL)^#^	36.00 (28.40, 43.00)	23.80 (26.50, 39.70)	0.019^**∗**^

PD: Parkinson's disease; UPDRS: Unified Parkinson's Disease Rating Scale; modified HY-stage: modified Hoehn Yahr staging scale; SE-ADL: Schwab and England Activities of Daily Living Scale; MMSE: Mini-Mental State Examination.

^*∗*^ *P* < 0.05, PD patients versus controls.

^#^Median (IQR): IQR: interquartile range.

**Table 2:** Correlation analysis of levels of total apoptotic leukocytes and their subpopulations to brain metabolite levels measured by ^1^H-MRS in the PD group after controlling for age and sex.

	Variables	*r*	*P* value
Neutrophil APO 2.7	*Dominant basal ganglia*		
	Cho/Cre	0.313	0.020^**∗**^
	*Basal ganglia-cerebellum ratio*		
	NAA/Cre	0.299	0.027^**∗**^
	Cho/Cre	0.376	0.005^**∗**^

Lymphocyte APO 2.7	*Dominant basal ganglia*		
	Cho/Cre	0.366	0.006^**∗**^
	*Basal ganglia-cerebellum ratio*		
	Cho/Cre	0.422	0.001^**∗**^

Total leukocyte APO 2.7	*Dominant basal ganglia*		
	Cho/Cre	0.277	0.041^**∗**^
	*Basal ganglia-cerebellum ratio*		
	Cho/Cre	0.271	0.045^**∗**^

Basal ganglia-cerebellum ratio, metabolite value in the dominant basal ganglia divided by the value in the cerebellum; PD: Parkinson's disease.

^**∗**^ *P* < 0.05, PD patients versus controls.

**Table 3:** Correlation analysis between plasma nuclear DNA to brain metabolite levels measured by ^1^H-MRS in the PD group after controlling for age and sex.

	Variables	*r*	*P* value
Plasma nuclear DNA	*Dominant basal ganglia*		
	Cho/Cre	0.277	0.069

Plasma nuclear DNA	*Cerebellum*		
	NAA/Cre	–0.190	0.218
	NAA/Cho	–0.033	0.831
	NAA/Cho + Cre	–0.130	0.400

Plasma nuclear DNA	*Basal ganglia-cerebellum ratio*		
	NAA/Cre	0.284	0.061
	Cho/Cre	0.384	0.010^**∗**^

Basal ganglia-cerebellum ratio, metabolite value in the dominant basal ganglia divided by the value in the cerebellum; PD: Parkinson's disease.

^*∗*^ *P* < 0.05, PD patients versus controls.

## Abbreviations

PD: Parkinson's disease
MRS: Magnetic resonance spectroscopy
DNA: Deoxyribonucleic acid
NAA: N-Acetylaspartate
Cre: Creatinine
Cho: Choline
BBB: Blood brain barrier
MMSE: Mini-Mental State Examination
UPDRS: Unified Parkinson's Disease Rating Scale
HY-stage: Hoehn and Yahr stages
SE-ADL: Schwab and England Activities of Daily Living Scale
PRESS: Point resolved spectroscopy sequences
TR: Repetition time
TE: Echo time
VOI: Voxel of interest
FWHM: Full width at half maximum
SD: Standard deviation
ANCOVA: Analysis of covariance (ANCOVA)
NMDA: N-Methyl-D-aspartate
LPS: Lipopolysaccharide.
